# Identification of Pyrrolizidine Alkaloids in *Senecio* Plants by Liquid Chromatography-Mass Spectrometry

**DOI:** 10.1155/2021/1957863

**Published:** 2021-11-16

**Authors:** An-Jing Lu, Yan-Liu Lu, Dao-Peng Tan, Lin Qin, Hua Ling, Chang-Hong Wang, Yu-Qi He

**Affiliations:** ^1^Key Laboratory of Basic Pharmacology of Ministry of Education and Joint International Research Laboratory of Ethnomedicine of Ministry of Education, School of Pharmacy, Zunyi Medical University, Zunyi, Guizhou 563000, China; ^2^School of Pharmacy, Georgia Campus-Philadelphia College of Osteopathic Medicine, 625 Old Peachtree Rd NW, Suwanee, GA 30024, USA; ^3^Shanghai Key Laboratory of Complex Prescription, Shanghai University of Traditional Chinese Medicine, 1200 Cai-Lun Road, Shanghai 201203, China

## Abstract

Pyrrolizidine alkaloids (PAs) are considered as the major constituents that cause hepatoxicity in *Senecio* plants. PAs can be found in about 3%–5% of the world's flowering plants. Nowadays, the identification method of PAs by separation and preparation was too slow and lacked effective power. A rapid method to identify PAs in plants must be developed. Based on the fragmentation regularity, the hepatoxic PAs and nonhepatoxic PAs were characterized by liquid chromatography-mass spectrometry (LC-MS). The detailed structures of PAs in five *Senecio* plants were identified based on tandem mass spectrometry (MS/MS) spectrum and chemical research information. In the present study, some new fragmentation regularities of PAs have been found, such as product ions at *m/z* 122, *m/z* 140 and *m/z* 124, *m/z* 142, which have been discovered as the characteristic fragments of lactone and mono-esterase type of saturated PAs, respectively. Moreover, two product ions at *m/z* 120 and *m/z* 138 have been reported as the characteristic fragments of unsaturated PAs. Some of them were found in *Senecio* species for the first time, and some of them may be new nature product or even new compound. Finally, we classified these plants into five categories based on PAs which were identified in the present study; the result corresponded with the classification by morphology. In addition, we have found some constituents that have odd molecular weight number only in *Senecio* species but not in *Ligularia* species; the detailed structures of these non-PAs constituents need penetrating study. LC-MS was rapid and sensitive method for detecting and identifying PAs in plants. Pyrrolizidine alkaloids were the toxiferous constituent of *Senecio* plants. In this study, we found that PAs can be used as the characteristic constituent of *Senecio* species.

## 1. Introduction

**Figure 1:**
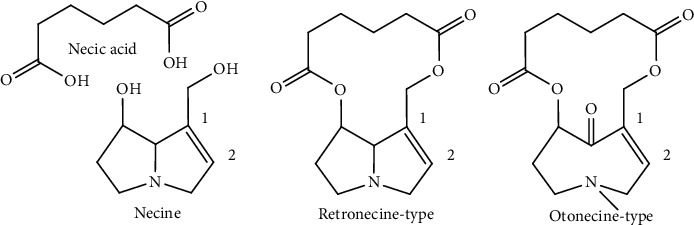
Structures of pyrrolizidine alkaloids.

In Traditional Chinese Medicine, *Senecio* plants were applied for thousands of years. In the world, about 3%–5% of all flowering plants contain PAs, which are one of the most toxic constituents in plants and cause hepatotoxicity mainly with the major clinical symptom of veno-occlusive disease (VOD) and other toxicities such as neurotoxicity, genotoxicity, et al. [1]. The mechanism of the toxicity of PAs is considered as metabolic activation; first, PAs was metabolized by P450 enzymes to generate dehydropyrrolizidine (DHP), then DHP with strong electrophilicity attacks proteins or DNA molecules to generate adducts, and finally, various signs of toxicity are induced [2]. This kind of compounds come from the esterification of carboxylic acid named necic acid and double pyrrole ring named necine and have been classified into two types named retronecine and otonecine, and 1,2-double bond is the key structure that causes toxicity (Figure 1). Because of the extensive distribution of PAs-containing plants, identification of the PAs in plants can prevent people from toxicity of PAs effectively.

Methods for analyzing PAs have been developed in this field for many years including thin-layer chromatography (TLC), high-performance liquid chromatography (HPLC), gas chromatography (GC), gas chromatography-mass spectrometry (GC-MS), and LC-MS. The lack of chromophores in PAs structures results in no strong UV absorption. As a result, the limit of detection (LOD) of ultraviolet (UV) detector is so high [3] that HPLC coupled with only PDA detectors can hardly detect the PAs in plants. Derivatization can add chromophores to the structure of PAs, making the absorption peak of PAs shifting to vision range from UV range and increasing the response for many times; however, this method transfers various PAs to the same product that cannot reflect individual structure of PAs, so this method is only applied for the determination of total PAs [4]. GC-MS is a new method that has high sensitivity, but high gasification temperature may cause decomposition of sample. In recent years, LC-MS has been applied to determinate the constituents of plants [5]. This analysis method overcomes possess lower LOD and can get structure and molecular weight information. Combined with the chemical research of plants, identification by LC-MS technology can conclude the structure of the constituents in plant samples. LC-MS has the function of superior sensitivity and specificity in both the chromatographic separation and detection steps. Traditional methods of detection (UV) and nuclear magnetic resonance (NMR) cannot offer sufficient sensitivity and specificity needed. Studies of the fragment regularity of PAs and the determinations of PAs in various samples by LC-MS have been reported before. But there was no further progress that studied the detailed structure of PAs by LC-MS. In the present study, we have identified the detailed structure of PAs in five *Senecio* plants that belong to Compositae by LC-MS technology combined with the chemical research information of these five plants, which can be differentiated from each other based on the PAs constituents in the present study. Morphology research has confirmed that the LC-MS results is correct. We analyzed one extra *Ligulariopsis* plant that also belongs to Compositae: ten constituents were found in five *Senecio* plants but not in *Ligulariopsis* species plant. These constituents could be used as effective tags that differentiate the *Senecio* plants from other Compositae plants.

## 2. Materials and Methods

### 2.1. Samples and Reagents

The standard references including Adonifoline, Isoline, Clivorine, and Senkirkine were isolated by our lab from *Senecio scandens*, *Ligularia duciformis*, *Ligularia hodgsonii*, and *Senecio solidagineus*, respectively [6–9]. Monocrotaline, isolated from *Crotalaria assamica*, was kindly supplied by Professor Zhiben Tu (Wuhan Botanical Institute, Academia Sinica, Wuhan, China). Their structures were confirmed using UV, IR, NMR, and MS analysis, and HPLC analysis showed that the purity of all standard references was 98%. Among five *Senecio* plants, the herbs of *Senecio nemorensis*, *Senecio cannabifolius*, and *Senecio cannabifolius* var. *integrifolius* were collected from Jilin province. The herbs of *Senecio vulgaris* and *Senecio scandens* were collected from Heilongjiang province and Guangdong province, respectively. Moreover, the harvesting place of the herb *Ligularia duciformis* was Gansu province. All samples were identified by Dr. Lihong Wu and voucher specimens were deposited at the Herbarium of Shanghai R&D Center for Standardization of TCM. HPLC-grade acetonitrile was purchased from Sigma-Aldrich (St. Louis, MO, USA). HPLC-grade formic acid was purchased from Tedia Company (Fairfield, OH, USA). HPLC-grade water was produced with a Milli-Q water purifying system. All other reagents were purchased from commercial source.

### 2.2. Main Equipment

The analysis was performed using a Thermo Finnigan HPLC instrument equipped with a quaternary pump, a photodiode array detector (PDA), an autosampler, and a column compartment. The HPLC system coupled with an ion trap mass spectrometry of LCQ XP Plus via an Electrospray Ionization (ESI) interface for mass analysis and detection. Data was acquired by the Xcalibur software obtained from Thermo Finnigan. The centrifugal used in this experiment was Biofuge Primo.

### 2.3. HPLC Conditions

Chromatographic separation was performed on the Phenomenex Synergi MAX-RP C_12_ column (4 um, 250 × 4.6 mm) together with a supported C_12_ guard column. The mobile phase was consisted of solvent A (1% formic acid in water) and solvent B (acetonitrile); the eluting process was as follows: beginning from 5% B and with a gradient from 5% B to 28% B for 25 min, held at 28% B for 30 min, then to 95% for 10 min, held at this percentage for 10 min. The mobile phase flow rate was 1 mL/min; the column temperature was set at 25°C.

### 2.4. MS Parameters

All mass spectrometric experiments were performed on an ion trap mass spectrometer. The LC effluent was introduced into the ESI source in a post-column splitting ratio of 10 : 1. The MS detector was optimized to obtain maximum signal of [*M*+*H*]^−^ ions of isoline which was one of the retronecine-type pyrrolizidine alkaloids. Scan mode was positive. The optimal parameters were as follows: capillary Temp was 300°C, sheath gas flow, Aux/Sweep Gas Flow were 32 and 10, respectively (arbitrary unite), source voltage was 5 KV, source current was 80 *μ*A, and capillary voltage was 9 V. The HPLC conditions and MS parameters in this text were modified based on [10].

### 2.5. Sample Preparation

Each dried sample was ground into fine powder (40 mesh) using a pulverizer, and then 1g was soaked in 15 mL of 0.2% (m/v) hydrochloric acid followed by an ultrasonication for 40 min. Next, the liquid was centrifuged at 10000 × *g* for 5 min, and an aliquot of 10 mL supernatant was transferred to another centrifuge tube of 50 mL; 2 ml of ammonia water was added to improve the pH value of the system followed by adding 25 ml of chloroform. After the above procedure, liquid-liquid extraction was performed by vortex to extract the alkaloids from the aqueous phase and then the mixture was centrifuged at 10000 × *g* for 5 minutes to accelerate the delamination of the emulsion.

20 mL of lower liquid was transferred to a round-bottomed flask of 50 mL, and then the solvent was evaporated by rotary evaporation in a water bath at 40°C under reduced pressure. 2 mL of 0.2% (m/v) hydrochloric acid was added to the round-bottomed flask for dissolving the alkaloids after the drying procedure. Finally, the dissolved liquid was filtered with a filter paper of 0.3 um to form the final sample, and 10 uL of sample was used for LC-MS analysis. Except for five species plants including *Senecio nemorensis*, *Senecio vulgaris*, *Senecio cannabifolius*, *Senecio cannabifolius* var. *integrifolius*, and *Senecio scandens*, one *Ligularia* species plant has been also prepared as described above.

### 2.6. Direct Loop Inject Analysis

Pure PAs, which contains adonifoline, isoline, monocrotaline, clivorine and senkirkine, were dissolved in 0.2% (m/v) hydrochloric acid and analyzed by loop injection using MS and MS/MS with electrospray ionization. Each sample was tested in triplicate.

## 3. Results and Discussion

### 3.1. HPLC Separation

**Figure 2:**
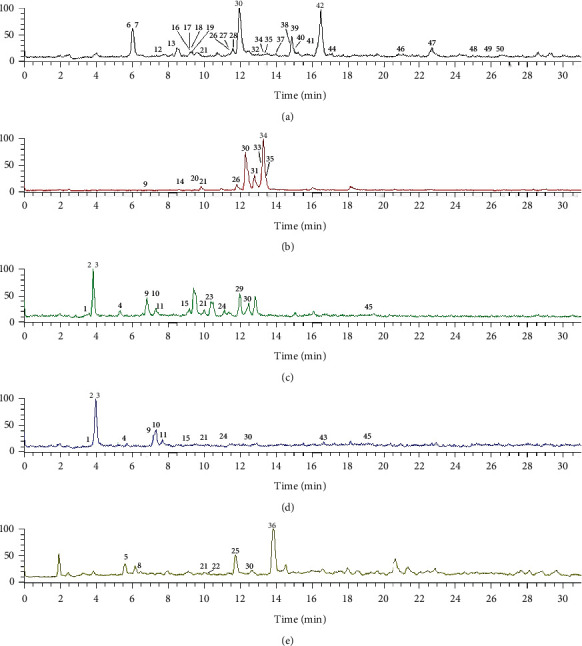
Total ion chromatograms (TIC) of *Senecio nemorensis* (a), *Senecio vulgaris* (b), *Senecio cannabifolius* (c), *Senecio cannabifolius* var. *integrifolius* (d), and *Senecio scandens* (e). For peak assignment, see Table 1.

Pyrrolizidine alkaloids are a class of nonpolarity compounds that that usually have difficulty in better separation under normal reversed-phase conditions. In order to improve the resolution and separation efficiency, C12 instead of C_18_ columns and formic acid with high concentration in the mobile phase were employed to improve the analytical method. Another reason was to increase the ionization efficiency in ESI source. The total ion chromatograms (TIC) of five *Senecio* species are shown in Figure 2. Since the content of PAs in plants is very low, most peaks of this kind of compound could not be observed in TIC. However, we can obtain many peaks in selective ion monitoring (SIM) mode, further, to identify them with multiple mass fragments based on fragmentation regularity, which was obtained from standard substance, and chemical investigation of related plants.

### 3.2. Fragmentation Regularity

**Scheme 1:**
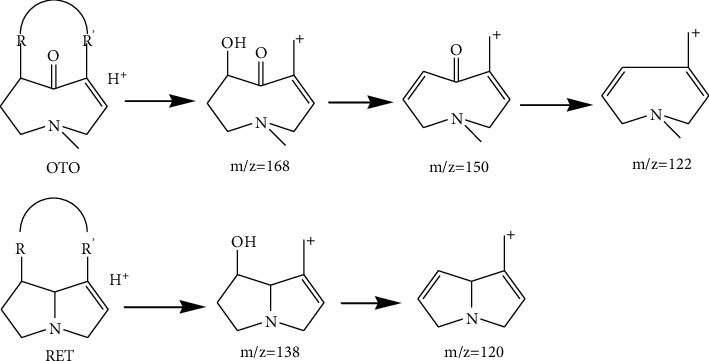
Mass fragment regularity of different types of PAs that have been reported.

**Scheme 2:**
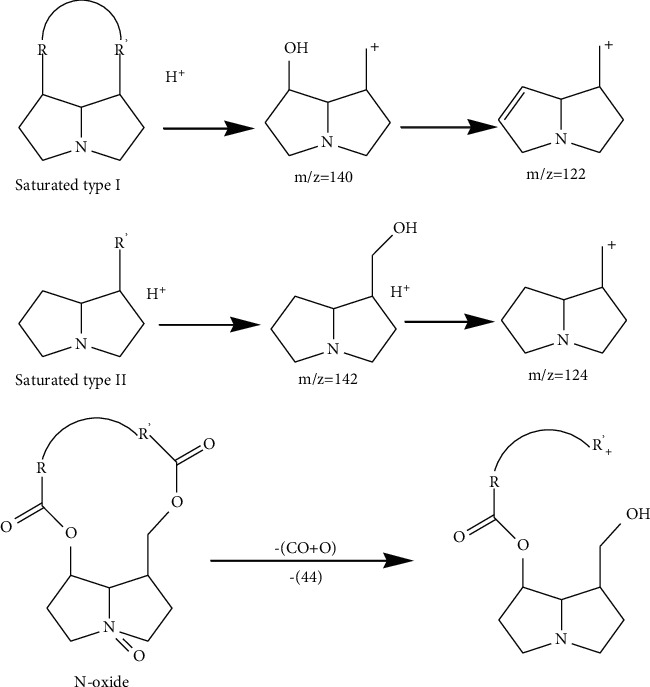
Mass fragment regularity of PAs that have been found in our experiment.

To demonstrate the characteristic of mass fragment, we analyzed five pyrrolizidine alkaloids with loop injection method for MS/MS analysis; two of them were not tested before the present study. Pure compounds we used are isoline, monocrotaline, clivorine, adonifoline, and senkirkine, which were isolated by our lab, and the last two were analyzed for the first time. Two fragment ions at *m/z* 120 and *m/z* 138 from positive mode were observed as the most characteristic ions of retronecine-type PAs, where *m/z* 122, *m/z* 150, and *m/z* 168 were observed as the most characteristic ions of otonecine-type PAs. The regularity was consistent with the result of reported study [11] (Scheme 1). After identification of the structure of PAs in plants, we found some other regularity that can be concluded, though the data was not from the analysis of pure alkaloids. We found that (1) saturated structure at 1,2 site of retronecine-type may produce two characteristic fragment ions at *m/z* 122 and *m/z* 140; (2) further if there was no substitution at 7 site of saturated retronecine-type compounds, two fragment ions at *m/z* 124 and *m/z* 142 may be produced; (3) N-oxide structure of PAs may directly loss a fragment only consisted of an oxygen but coupled with a loss of CO; then a neutral fragment ion at *m/z* 44 may be produced (Scheme 2). We think these discoveries are very important, because whether 1,2 site is saturated correlates with the toxicity of PAs; unsaturated 1,2-bond is considered as the key structure of the PAs which are toxical, and N-oxide retronecine-type is one of the most important forms of nontoxic PAs [12]. This can explain why some plants, which contain considerable PAs and exist as a major constituent in some complex prescription of Traditional Chinese Medicine (TCM), do not show intended toxicity.

### 3.3. Strategy of Identification

Because of the lack of UV absorption, PAs could not be detected under PDA detector. Thus, all analyses were conducted without PDA. For ensuring which peak was PA, we designed a two-step procedure; first, we screen all the molecular, which can be found in TIC or SIM; then, the molecular weight of odd number was chosen to conduct MS_2_ fragment; second, we judged the structure feature based on the characteristic MS^2^ ion fragment from [*M*+*H*]^+^. For example, *m/z* 120 and *m/z* 138 indicated the peak was an unsaturated retronecine-type PAs, *m/z* 122 and *m/z* 140 indicated the peak was a saturated retronecine-type PAs, *m/z* 122, *m/z* 150, and *m/z* 168 indicated the peak was an unsaturated otonecine-type PAs. To confirm the detailed structure of each peak, except the characteristic fragment, other MS^2^ fragments were analyzed, such as [*M* + H–H_2_O] ^+^, [*M* + H-60] ^+^, [*M* + H-28] +  that were used to judge the substituting group; the odd number of fragments was used to judge whether the PA was a macro ring type or an open-chain type. Chemical research information also was used as main evidence for identification. When several structures matched the same molecular, the one that has been reported in *Senecio* species could be selected.

### 3.4. Identification of Pyrrolizidine Alkaloids in Five *Senecio* Species

**Figure 3:**
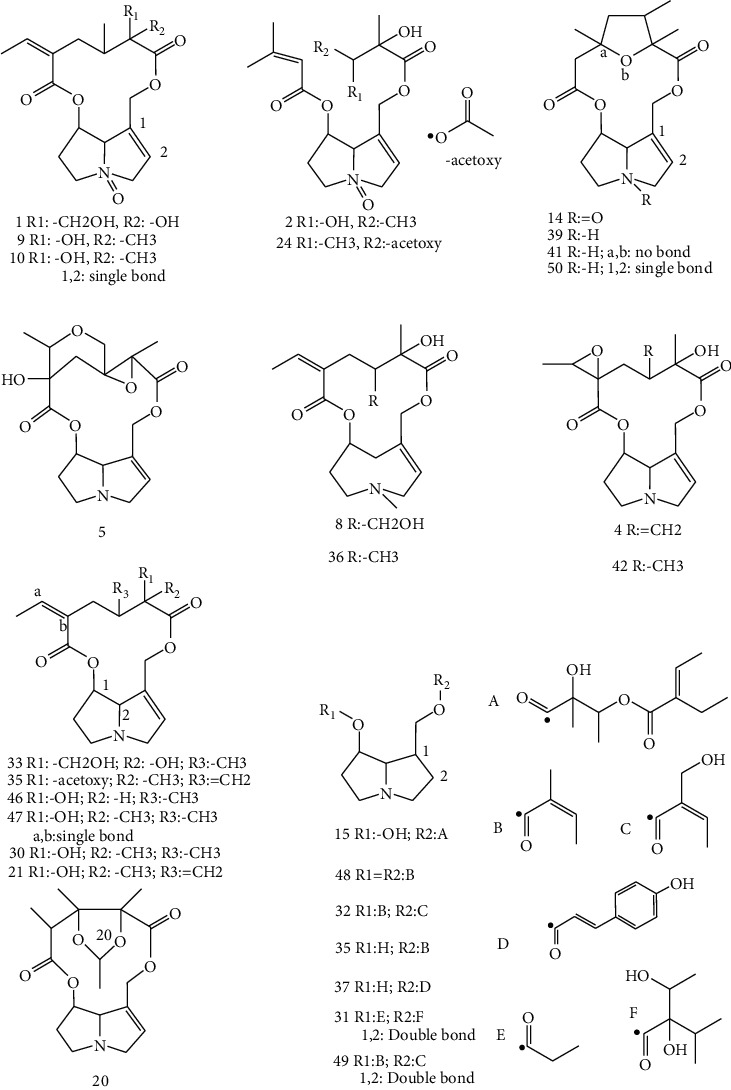
Structure of PAs that have been identified in five *Senecio* species.

Based on the regularity of mass fragment, 85 PAs have been detected by LC/MS/MS; 75 of these are retronecine-type and others are otonecine-type. The molecular and general structure type information are shown in Table 1. Among the 85 PAs, nearly 50 PAs were identified to recognize the detailed structure (Figure 3). Both open-chain structure and macro cycle structure have been identified in plants analyzed in this experiment. Saturated and unsaturated structures and N-oxide form of retronecine-type have been identified also. Some constituents of PAs have been found in specific species for the first time. Some PAs constituents exist in both near species, and this phenomenon corresponds with the morphologic plant-classification. Except for PAs, some other chemical constituents have been found in *Senecio* species but not in *Ligularia* species plant; these can be selected as the characteristic constituents to distinguish *Senecio* species from other species plant by chemical analysis method, which may be more useful than traditional method observed by eyes or nose used in Traditional Chinese Medicine (TCM) especially when the sample was powder.

Compound 21 was a common constituent in all five *Senecio* species that we tested in the present study. From the MS data, the compound at *m/z* 334 corresponding to [*M*+*H*]^+^ was achieved; from the chemical study, only two structures with the molecule weight of 333 were discovered in *Senecio* species. They are Leptanthine N-oxide and Seneciphylline, respectively. Furthermore, from the data of MS^2^ of the *m/z* 334, a series of characteristic fragment ions at *m/z* 120 and *m/z* 138 have been found in pyrrolizidine alkaloids. So, we can judge that this compound has an unsaturated structure at 1,2 sites. Comparing the two structures we got from another chemical study, seneciphylline was closer to this peak in all five species [13].

Compound 30 was another constituent existing in all five *Senecio* species. From the MS data, the quasimolecular ion of the compound at *m/z* 334 corresponding to [*M*+*H*]^+^ was obtained. From the literature about *Senecio* species chemicals, eight PAs have been found. Several evidences made the structure localized. First, from MS^2^ data of *m/z* 336, characteristic fragment ions at *m/z* 120 and *m/z* 138 have been found and there was no obvious neutral loss mass of 16, which was generated by N-oxide, existing in the MS^2^ spectrum, so the structure was a non-N-oxide and an unsaturated one. From chemical research, only one PA that named Senecionine, possessing a molecular weight of 335, exists in *Senecio vulgaris* [14]; in the present experiment, compound 30 also was the only one that has a quasimolecular ion of *m/z* 336, so we can judge that compound 30 was Senecionine.

Compound 39 was an obvious peak existing in *Senecio nemorensis* and has an [*M*+*H*]^+^ ion of *m/z* 336. In ESI-mode, if a molecule has hydroxyls, water molecules may be lost from [*M*+*H*]^+^ ion easily and generate an ion of [*M* + H-18]^+^. But this type of mass fragment has not been found in the MS^2^ spectrum of compound 39. In all of the compounds discovered in *Senecio* species and which possess a molecular weight of 335, only retroisosenine has no hydroxyls at whole molecule. So, retroisosenine was the closest structure to compound 39 [15].

**Figure 4:**
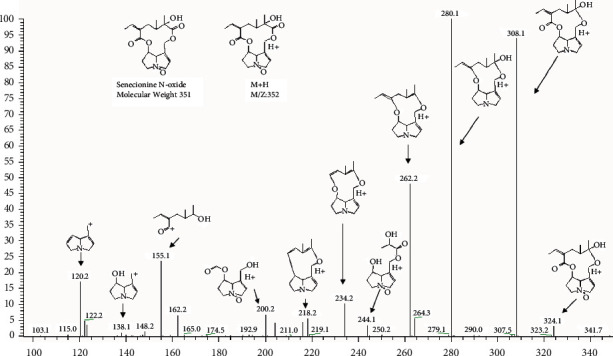
MS2 spectrum and fragment of compound 9: Senecionine N-oxide.

Compound 9 was a peak existing in *Senecio cannabifolius*, *Senecio cannabifolius* var. *integrifolius*, and *Senecio vulgaris*. The MS data showed that the quasimolecular ion mass was *m/z* 352. The MS^2^ fragment ion at *m/z* 120 and *m/z* 138 prompted that the structure was retronecine-type and unsaturated at 1,2 site. The appearable peak of [*M* + H-44]^+^ may be because of [*M* + H–CO_2_]^+^ or [*M* + H–CO–O]^+^; in this kind of compound, there was hardly a radical that could generate the neutral fragment of CO_2_, so [*M* + H–CO–O]^+^ was the most possible. To our presumption, an oxygen atom existing in a special state must be included in the possible structure. [*M* + H-18]^+^ showed that there was one or more hydroxyl at the structure to generate the peak which came from a water molecule loss. Sixteen structures of pyrrolizidine alkaloids, having a molecular weight of 351, were discovered by the chemical study of *Senecio* species. Several otonecine-type structures failed to correspond with compound 9. Certain structures that were substituted by free radicals at the pyrrole ring also failed to match compound 9, because substitution at pyrrole ring could alter the characteristic ion mass number. Compounds of N-oxide and three rounds epoxy just had an oxygen atom can be lost alone and may generate the [*M* + H–CO–O]^+^, so the probable structure should be N-oxide or the one with three rounds epoxy. Then, only three structures could correspond with the MS^2^ spectrum of compound 9; two of them were N-oxide: one is open chain and the other is macro cycle, and the other one with three rounds epoxy. Finally, two fragment ions at *m/z* 115 and *m/z* 155 had been found in MS^2^ spectrum; from the structure of the open chain one, the acid part could not generate as large fragment ions at *m/z* 115 and *m/z* 155. So, the macro cycle one was closer. Considering the chemical study of *Senecio cannabifolius*, Senecionine N-oxide had been separated from this plant, so the final structure was Senecionine N-oxide (Figure 4) [16].

Compound 35 is also owned by both *Senecio nemorensis* and *Senecio vulgaris*; its molecular weight is 223. From MS^2^ spectrum, the characteristic fragment ions at *m/z* 124 and *m/z* 142 indicated that the type of this compound was saturated and singly substituted at site 7 or site 9. From reported data, Isoretronecyl tiglate [17] has separated from *Senecio* plants just corresponds to this feature, so we conclude compound is Isoretronecyl tiglate. Furthermore, there is no hydroxyl radical in this molecular, so there must be no [*M* + H-18]^+^ found in MS^2^ spectrum; actually, there is indeed no *m/z* 206 occuring in MS^2^ spectrum.

Compound 1 is owned by both *Senecio cannabifolius* and *Senecio cannabifolius* var. *integrifolius.* Molecular weight is 367.6. Among these compounds reported from *Senecio* plants, crosemperine and dihydrosenkirkine cannot generate the characteristic fragments of *m/z* 120 and *m/z* 138 because their structures were otonecine-type. Casuarine 6-D-glucoside and crotaflorine also cannot generate the two fragments because of core structure. So, the most probable structure must like remain two named spectabilin and retrorsine N-oxide. According to the structure of spectabilin, substitution of ethyl ester at 12 sites must generate a fragment of [*M* + H-60]^+^, but there is no fragment possessing the m/*z* of [*M* + H-60] + in MS^2^ spectrum of compound 1. So, retrorsine N-oxide is the most probable structure of compound 1 [18].

Compound 3 is another common constituent of *Senecio cannabifolius* and *Senecio cannabifolius* var. *integrifolius.* 6 pyrrolizidine alkaloids possessing the same molecular weight as compound 3 have been reported; among them, adonifoline was removed from the probable structure because its retention time is not the same as compound 3. MS^2^ spectrum indicated this compound is retronecine-type, so senkirkine and emiline have been removed out. Finally, because Seneciocannabine [19] had been reported in *Senecio cannabifolius*, so it must be the most probable structure of compound 3.

Compound 10 has the molecular weight of 353; there are 18 structures, which have been reported in *Senecio* plants, corresponding with this molecular weight. MS^2^ spectrum showed that compound 10 is a saturated one because of the fragments of *m/z* 122 and *m/z* 140. So, 9 unsaturated PAs have been moved out from candidates. Furthermore, the one that was substituted in pyrrole ring was also moved out. Because [*M* + H-44]^+^ occurs in MS^2^ spectrum of compound 10, so it may be an N-oxide or a three rounds epoxy. Odd fragment of *m/z* 169 indicated that compound 10 is not an open-chain structure. From MS^2^ spectrum, we found that there is no hydroxyl radical substituted at molecular. Accumulating all above pieces of information, oxynemorensine [20] is the most probable structure of compound 10.

Compounds 11, 15, 24, and 45 also are owned by both *Senecio cannabifolius* and *Senecio cannabifolius* var. *integrifolius*. Judged from molecular weight, MS^2^ spectrum, fragment regularity, and chemical research data, we concluded the structures of these four compounds as Jaconine, Ipanguline 3″-Tigloyl, Retronecine 9-(3-acetoxy-2-hydroxy-2-methylbutanoate) 7-Senecioate N-oxide, and Seneciphyllinine. For compound 11, because of loss of neutral fragment of 36 that can be considered as HCl, the structures of jaconine and Merenskine, which are different from each other on only a methyl radical, are very close to compound 11. For compound 15, fragments of *m/z* 122 and *m/z* 140 indicated that it is a saturated compound, and both site 7 and site 9 have been substituted; among the reported structures which have a molecular weight of 355, two compounds, named Ipanguline 2″-Tigloyl and Ipanguline 3″-Tigloyl, corresponded with the feature of compound 15. For compound 24, because [*M* + H-60]^+^ occurs in MS^2^ spectrum, so Retronecine 9-(3-acetoxy-2-hydroxy-2-methylbutanoate) 7-*Senecio*ate N-oxide and O-acetylcholine both correspond with this compound, but odd fragment of *m/z* 193 indicated that open-chain structure was impossible, so O-acetylcholine is the closest structure to compound 24. For compound 45, fragments of *m/z* 120 and *m/z* 138 indicate it is a unsaturated compound, while fragment of *m/z* 316 indicates there is a ethyl ester substituted at the structure; among three candidates, Seneciphyllinine [21] is the closest one.

For *Senecio* scandens, Adonifoline [22] is the characteristic constituent; by comparing the retention time and MS^2^ spectrum, we judged that compound 5 is adonifoline. And compound 36 has the same molecular weight as adonifoline, but fragments of *m/z* 122, *m/z* 150 and *m/z* 168 indicate that compound 36 is otonecine-type PAs, combined the MS^2^ spectrum and chemical study data, senkirkine [23] was considered as the closest candidate. Other peaks have been identified for their detailed structures; data is summarized in Figure 3.

### 3.5. Probable Structures of Several Peaks

**Figure 5:**
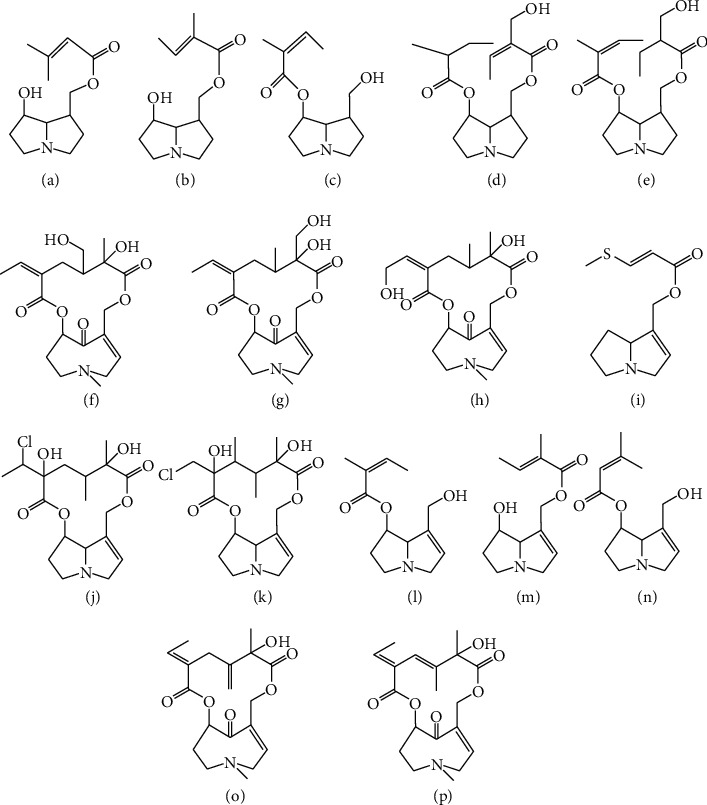
Probable structures of several peaks. (a) Fuchsisenecionine. (b) Racemocine. (c) 7-Angeloylturneforcidine. (d) Dihydro-Sarracine. (e) Dihydro-Sarracine. (f) 18-Hydroxysenkirkine. (g) Hydroxysen kirkine. (h) Anonamine. (i) Planchonelline. (j) Jaconine. (k) Merenskine. (l) 7-Angeloylretronecine. (m) 9-Angeloylretronecine. (n) 7-Senecioylretronecine. (o) Dehydro-senkirkine I. (p) Dehydro-senkirkine II.

Compounds 22 and 25 have the same molecular weight of 363; characteristic fragment ions at *m/z* 122, *m/z* 150, and *m/z* 168 indicated the two compounds were otonecine-type, and their MS^2^ spectrums were the same. But there was no structure that can correspond with the feature of compounds 22 and 25. Consider that the RT of compounds 22 and 25 were 10.02 min and 11.85 min, which were very close to the RT 13.83 min of senkirkine, and all of their structures were otonecine-type, the molecular weights of compounds 22 and 25 were 363, which were 2 Da smaller than senkirkine only, so we conclude that these two compounds were two dehydrated compounds of senkirkine; here we named them dehydro-senkirkine I and dehydro-senkirkine II. From the structure of senkirkine, we found that only two bonds could be dehydrated. So, the most probable structure of compounds 22 and 25 was shown in Figures 5(o) and 5(p). The two structures had not been reported before.

Compounds 7, 18, and 28 also had the same MS^2^ spectrum and molecular weight. But only one known structure can correspond with these spectrums. Fragments of *m/z* 124 and *m/z* 142 indicated structures were saturated and there was no radical substituted at site 7 or site 9; according to these pieces of information, planchonelline [24] just has the closest structure. But three peaks have the same feature, so we concluded that these three compounds were isomers and have the closest structure to planchonelline. For the same reason, we concluded that compounds 6, 17, and 27 were also isomers, and the most probable structure is fuchsiasenecionine [25] because this compound had been reported in *Senecio nemorensis*. Furthermore, we can recommend several structures, which were 7-Angleoylturneforcidine, 7-Angeloylplatynecine, 9-Angeloylplatynecine, and Racemocine, for these three isomers.

**Figure 6:**
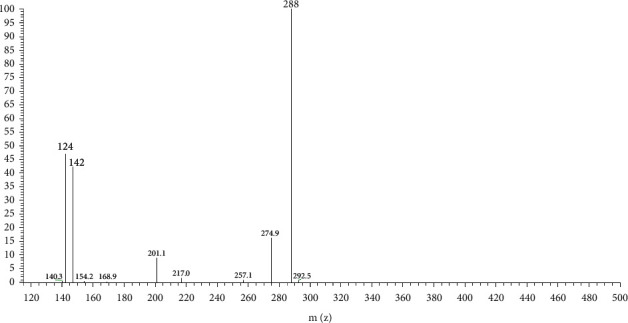
MS2 spectrum of compounds 26 and 34, probable new compounds.

Compounds 26 and 34 are owned by both *Senecio nemorensis* and *Senecio vulgaris* whose molecular weight is 433; two typical fragments of *m/z* 124 and *m/z* 142 indicated that the two constituents were saturated PAs and were substituted at only site 7 or site 9 (Figure 6). But we cannot conclude the detailed structure because of the lack of chemical research data. Because the MS^2^ spectra were very similar, these two constituents may be two isomers. To our knowledge, there was no other pyrrolizidine alkaloid that had the same molecular weight reported. Thus, we concluded that the two compounds were new compounds. All probable structures we concluded are shown in Table 1 and Figure 5.

### 3.6. Comparison of Non-PAs Constituents between *Senecio* and *Ligularia* Species

**Figure 7:**
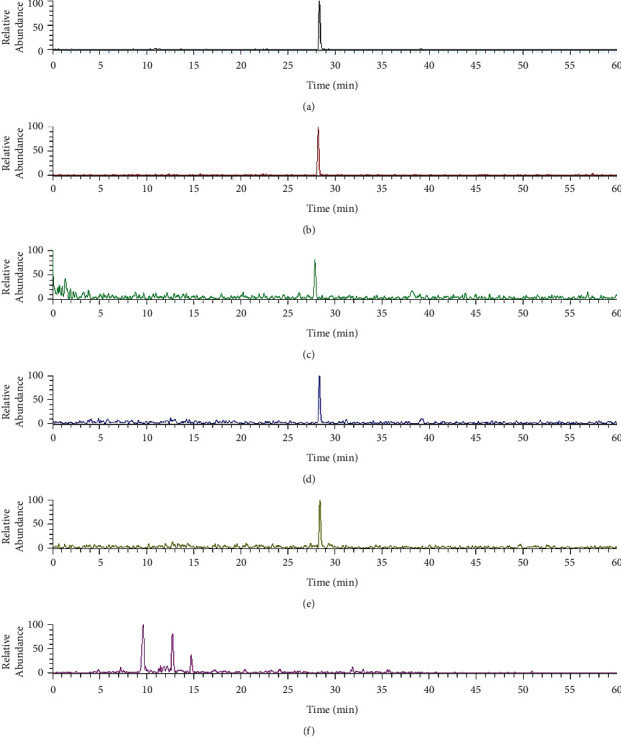
SIM of 246 in (a) Senecio nemorensis, (b) Senecio vulgaris, (c) Senecio cannabifolius, (d) Senecio cannabifolius var. integrifolius, (e) Senecio scandens, and (f) Ligularia duciformis.

Except pyrrolizidine alkaloids, other constituents had been found in all five *Senecio* species, although we cannot identify their structure, some regularity existed about these constituents. These constituents were contained in all five *Senecio* species but could not be found in *Ligularia* species, so these constituents could be used to distinguish *Senecio* species from others. Series [*M*+*H*]^+^ of them were *m/z* 272, *m/z* 282, *m/z* 394, *m/z* 408, *m/z* 424, *m/z* 238, *m/z* 254, *m/z* 436, *m/z* 438, *m/z* 440, *m/z* 450, *m/z* 458, *m/z* 446, *m/z* 470, *m/z* 474, *m/z* 492, *m/z* 218, *m/z* 302, *m/z* 312, *m/z* 314, *m/z* 266, *m/z* 318, *m/z* 334, *m/z* 338, *m/z* 340, *m/z* 410, *m/z* 484, *m/z* 362, *m/z* 580, *m/z* 486, *m/z* 328, *m/z* 330, *m/z* 342, *m/z* 344, *m/z* 346, *m/z* 360, *m/z* 364, *m/z* 378, *m/z* 380, *m/z* 482, *m/z* 228, *m/z* 218, *m/z* 290, *m/z* 244, *m/z* 266, *m/z* 502, *m/z* 590, *m/z* 526, *m/z* 496, *m/z* 490, *m/z* 350, *m/z* 422, *m/z* 452, *m/z* 316, et al. The SIM of *m/z* 246 is shown in Figure 7.

## 4. Conclusion

In the present work, we detected 85 PAs, 75 of these were retronecine-type and the other 10 were otonecine-type. Near 50 peaks among these were identified for detailed structures. Most of them have been reported before. But several of these were identified for the first time, for example, “dehydro-senkirkine.” Among all the constituents, compounds 26 and 34 may be new compounds. However, several compounds have not been identified because of the lack of chemical research information and some constituents were too low to respond so that the MS^2^ has not any signal.

LC-MS was rapid and sensitive method for detecting and identifying PAs in plants. In this study, we detected and identified more than 80 compounds in five *Senecio* plants; almost they cannot be detected by UV detector. So, in the future, LC-MS will still be the major method.

Pyrrolizidine alkaloids were the toxiferous constituent of *Senecio* plants; in this study, we found that PAs can be used as the characteristic constituent of *Senecio* species. For example, adonifoline can differentiate *Senecio scandens* from other four *Senecio* plants in our study. Most constituents were similar in *Senecio cannabifolius* and *Senecio cannabifolius* var. *integrifolius,* the latter was a variety of the former. Furthermore, we used other constituents, which have the odd molecular weight number to distinguish *Senecio* plants from *Ligularia* species; however, we cannot identify their structure; the merely available information was that these constituents may be another kind of alkaloids.

## Figures and Tables

**Table 1:** Characterization of major compounds from five *Senecio* species.

No.	RT (min)	[*M*+*H*]^+^	Compounds	Characteristic Fragment	Structure type	Main fragments
*Characterized components*						
1	3.62	368	Retrorsine N-oxide	120, 138	RET, US	120, 138, 238, 296, 324, 340
2	3.88	370	Retronecine 9-(2,3-dihydroxy- 2-metrylbutanoate)7-senecioate N-oxide	120, 138	RET, US	120, 138, 280, 308, 326, 342
3	3.95	366	Seneciocannabine	120, 139	RET, US	120, 138, 214, 276, 294, 322
4	5.35	350	Jacozine	120, 138	RET, US	120, 138, 212, 242, 260, 278, 306, 322
5	5.62	366	Adonifoline	120, 138	RET, US	120, 138, 338
8	6.61	382	18-Hydroxysenkirkine	122, 150, 168	OTO	122, 150, 168, 186, 268, 364
9	6.84	352	Senecionine N-oxide	120, 138	RET, US	120, 138, 200, 234, 262, 280, 308, 324
10	6.96	354	Platyphylline N-oxide	122, 140	RET, S	122, 140, 158, 169, 282, 310
14	8.76	352	Oxyretroisosenine	120, 138	RET, US	120, 138, 324, 352, 360
15	9.19	356	Ipanguline 3″-Tigloyl	122, 140	RET, S	122, 140, 310, 328
20	9.92	352	Monocrotalinine	120, 138	RET, US	120, 138, 156, 278, 322
21	9.96	334	Seneciphylline	120, 138	RET, US	120, 138, 288, 306
24	11.30	412	Retronecine 9-(3-acetoxy-2-hydroxy-2-methylbutanoate)7*-*senecioate N-oxide	120, 138	RET, US	120, 138, 262, 308, 352, 368, 384
30	12.45	336	Senecionine	120, 138	RET, US	120, 138, 290, 308
31	12.49	356	7-Propionylintermedine	122, 140	RET, S	122, 140, 222, 240, 256, 312
32	12.84	338	Sarracine	122, 140	RET, S	122, 140, 292, 310, 320
33	13.26	352	Retrorsine	120, 138	RET, US	120, 138, 220, 246, 324
35	13.53	224	Isoretronecyl tiglate	124, 142	RET, S2	124, 142, 224
36	13.83	366	Senkirkine	122, 150, 168	OTO	122, 150, 168
37	13.98	288	Thesinine	124, 142	RET, S2	124, 142, 170, 188
39	15.04	336	Retroisosenine	120, 138	RET, US	120, 138, 220, 254
41	16.14	336	Doronenine	120, 138	RET, US	120, 138, 220, 238, 254
42	16.37	352	Jacobine	120, 138	RET, US	120, 138, 237, 254, 334
45	19.09	376	Seneciphyllinine	120, 138	RET, US	120, 138, 288, 316
46	20.97	322	Nilgirine	120, 138	RET, US	120, 138, 154, 218
47	21.82	338	Yamataimine	120, 138	RET, US	120, 138, 220, 238, 256
48	25.00	322	Diangeloylplatynecine	122, 140	RET, S	122, 140, 222, 240
49	25.00	336	Triangularine	120, 138	RET, US	120, 138, 218, 236, 254
50	25.77	338	Nemorensine	122, 140	RET, S	122, 140, 220, 238, 256

*Tentatively characterized components*						
6	6.07	240	Fuchsisenecionine	122, 140	RET, S	122, 140, 158, 222
17	9.28					
27	12.00					

7	6.07	242	Planchonelline	124, 142	RET, S2	124, 142, 160
18	9.28					
28	12.00					

22	10.02	364	Dehydro-senkirkine	122, 150, 168	OTO	122, 150, 168, 318, 336, 346
25	11.85					

13	8.38	238	7-Angeloylretronecine	120, 138	RET, US	94, 120, 138, 156
16	9.21					

11	7.46	388	Jaconine or merenskine	120, 138	RET, US	120, 138, 324, 352, 360
12	7.76	336	Unknown	122, 140	RET, S	122, 140, 167, 222, 265
19	9.57	382	Otonecine or senecioracenine or petasitenine	122, 150, 168	OTO	122, 150, 168, 200, 240, 310, 338, 354
23	10.35	350	Seneciphylline N-oxide	120, 138	RET, US	120, 138, 246, 288, 322, 332
26	11.99	434	Unknow (new in nature product)	124, 142	RET, S2	124, 142, 288
34	13.45					

29	12.41	340	Unknown (dihydro-sarracine)	122, 140	RET, S	122, 140, 294, 312
43	16.41	340	Unknown	122, 140	RET, S	122, 140, 155, 322, 338
38	14.57	338	Bulgarsenine	122, 140	RET, S	
			Hastacine			
			Platyphylline			

RET: retronecine type; OTO: otonecine type; S: saturated type; S2: saturated type II and US: unsaturated type.

## Data Availability

All the data related to these findings are included in the manuscript.
